# Framing Effects on Willingness and Perceptions towards COVID-19 Vaccination among University Students in Italy: An Exploratory Study

**DOI:** 10.3390/vaccines11061079

**Published:** 2023-06-09

**Authors:** Massimo Cesareo, Marco Tagliabue, Magdalena Edyta Lopes, Paolo Moderato

**Affiliations:** 1 Istituto Europeo per lo Studio del Comportamento Umano (IESCUM), Piazzale Ravenet 5, 43100 Parma, Italy; dott.massimo.cesareo@gmail.com (M.C.);; 2 Department of Behavioural Sciences, Faculty of Health Sciences, OsloMet—Oslo Metropolitan University, St. Olavs Plass, P.O. Box 4, 0130 Oslo, Norway; 3 Department of Business, Law, Economics and Consumer Behaviour, IULM University, Via Carlo Bo 1, 20143 Milano, Italy

**Keywords:** vaccine hesitancy, framing, COVID-19, communication campaign, attitudes

## Abstract

Vaccine hesitancy is a diffused psychological phenomenon that has been increasingly addressed in several studies since the COVID-19 pandemic. Communication campaigns play a pivotal role influencing recipients’ perceptions and may affect the likelihood to vaccinate or to show hesitancy. In the context of communicating risks during the COVID-19 pandemic, we hypothesized that highlighting different aspects of data on the effectiveness of vaccines would influence people’s willingness and attitudes to vaccinate. In this exploratory study, we administered two versions of a survey to a convenience sample of students from three universities in Italy. In the first version, salience was placed on the effectiveness of the vaccine in terms of reducing the probability of infection. In the second version, salience was placed on the effectiveness of the vaccine in terms of reducing the probability of hospitalization after being infected by COVID-19. The results confirmed our hypothesis: participants reported that they were more willing to become vaccinated when exposed to the hospitalization frame (main dimension). Conversely, we found mixed effects of the frame on the following sub-dimensions: reliability, trust, protection, safety, and confidence. Taken together, we show that it is possible to influence, to some extent, university students’ attitudes and perceptions toward COVID-19 vaccination by acting on how information is framed. We discuss the implications of these findings for the development of behaviorally informed policies.

## 1. Introduction

Vaccine hesitancy is a diffused psychological and emotional state, whose effect on COVID-19 vaccination rates has been recorded in several studies, e.g., [1,2]. It refers to a “behavioural delay in acceptance, or refusal of vaccines despite availability of vaccine services” [3]. Vaccine hesitancy was included by the WHO in 2019 among the ten greatest global health threats [4]. As of June 2021, 75.2% of 23,000 national representative respondents of 23 countries reported vaccine acceptance in the context of the COVID-19 pandemic [5]. Although these numbers look optimistic, there is still ample margin for the improvement of current and future vaccination campaigns for achieving public health goals, especially among youths with higher education. Barello et al. ran a cross-sectional design survey to a sample of Italian university students and found that about one out of ten respondents showed vaccine hesitancy and no or low intention to vaccinate [6].

In another study, hesitancy was reflected in negative attitudes among some segments of the worldwide population since the first vaccines to fight the COVID-19 pandemic were created, tested, and administered, based on either country or regional policies and laws; for example, they were influenced by political attitudes [7]. Several factors have been linked to vaccine hesitancy, which is pervasive phenomenon, including the heuristic of balancing risks and benefits and evaluating the success of vaccinating, whether it is unnatural, the degree of public confidence, and what others see [8].

In some cases, vaccine hesitancy has been regarded as being linked to the psychological underpinnings of confidence, complacency, and convenience [9]. In others, hesitancy led to slower public health protection, as well as difficulty in reaching the herd immunity threshold, thus weighing more heavily on the economic and social costs of lockdowns, quarantine and isolation, schooling, etc., e.g., [2,10,11]. Either way, it seems clear that communication campaigns about, among other things, risks and benefits of vaccinating, can affect vaccine hesitancy among recipients across population segments. For example, Marcell et al. [12] addressed pregnant people with their social media campaign “One Vax, Two Lives”, which was created to promote scientific risk communication of COVID-19 during pregnancy and the benefits of vaccinating. Moreover, the authors of that study provided a blueprint for addressing vaccine hesitancy through their initial findings among students to address other target groups, too. In another example, Hong reported the successful introduction of a social marketing approach in the mass vaccination campaign ran by the South Korean government that led to a high vaccine coverage [13].

Communication campaigns should target evidence-based levers among the population and use argumentation tools with experts; together with restoring behavioral insights of making vaccines accessible and spreading communication among early adopters, they address vaccine hesitancy and may increase vaccination rates [14]. For example, reducing vaccine hesitancy has been previously inquired by means of resorting to altruistic motivation (i.e., promoting altruism in the context of influenza vaccination) [15]. In a systematic review of peer-reviewed studies and grey studies, multicomponent and dialogue-based interventions were the most effective strategies for addressing vaccine hesitancy: these included raising knowledge and awareness in high-, as well as in low-income regions [16]. Although the COVID-19 pandemic boosted research and shed light on campaigns to counteract vaccine hesitancy, there is limited evidence to recommend any specific intervention against vaccine hesitancy [17] or parental vaccine refusal [18].

Communication campaigns, including mass- and social media, play a crucial role in the information of benefits and risks and bear a considerable responsibility toward the likelihood of developing and strengthening vaccine hesitancy among the public. Previous research has shown that how the same information is communicated can influence our choices and decisions, although the modality should not affect agents’ preferences [19]. The underlying assumption is that agents are rational: they have stable and ordered preferences (i.e., transitive and complete). The modality through which information is communicated, and not its content, are referred to as frames, which comprise the main variables of interest in the present study. Specifically, we are interested in how information framing affects willingness and perceptions toward COVID-19 vaccination among university students in Italy. Previous studies have addressed vaccination intentions and attitudes among university students in Italy [20,21] and in other European countries (e.g., Germany [22]; see also [23] for a review).

Some of the risks associated with taking any of the COVID-19 vaccines approved by the EU Commission are known and disclosed regardless of the type of vaccine. Some vaccines involve messenger RNA (m-RNA), which gives instructions to the body cells without conveying the actual virus (e.g., Moderna, Pfizer-BioNTech), while others involve vectors, where the COVID-19 virus’ genetic material is passed to the cells via another virus that serves as a vector (e.g., Janssen/Johnson & Johnson and AstraZeneca) [24]. Side effects include headache, fatigue, muscle and joint pain, fever and chills, and pain at the site of injection [25]. Other risks occur with a much lower incidence and have been explicitly reported to the vaccine’s type and/or producer.

In some countries, this led to blocking the administration of a specific vaccine to a particular gender or age group, recommending one vaccine type over another based on these characteristics, as well as letting users decide for themselves after disclosing this information or removing the vaccine type or brand from the national vaccination campaign. The latter resulted in a high degree of variability between European countries. For example, AstraZeneca’s vaccine was suspended in several European countries (e.g., Italy, Norway, Sweden, Germany—the latter pending approval from EMA) after some cases of thrombosis (blood clotting) were recorded among different segments of the population. Specifically, most of these cases occurred in women under 60 years of age within two weeks from vaccination and led to the conclusion that “unusual blood clots with low blood platelets should be listed as very rare side effects of Vaxzevria (formerly the COVID-19 Vaccine AstraZeneca)” [26].

### 1.1. Frames and Salience

Frames refer to the characteristics of information or messages. According to the rationality principle, e.g., [27], which subscribes to utility-maximizing agents and decision-makers, these characteristics should not influence choice. However, frames are related to salience, and there is strong empirical evidence that altering the characteristics of information or messages without changing its contents can change the response to a stimulus. This phenomenon is termed the framing effect. Framing effects have been observed in several domains, particularly medicine and health decision-making. Some examples related to the COVID-19 pandemic include framing vaccine resistance [28], communicating side effects [29], vaccination status discrimination [30], and vaccine acceptance in South India [31].

In the early 1980s, Tversky and Kahneman, e.g., [19,32], developed a stream of studies focusing on one of the principles described in neoclassical economics: invariance, according to which individuals’ preferences should not be affected by variations of irrelevant options or outcomes [32]. Notwithstanding, several empirical studies showed that altering how information is presented without altering its contents can have a significant impact on people’s decisions, e.g., [19], see also [33] for implications for citizen competence.

For example, in the “Asian disease” problem, developed by Tversky and Kahneman [19], two groups of participants were presented with the question of whether they would support one of two public health programs in the presence of a hypothetical disease outbreak. Program A was framed in terms of certain gains or losses of lives, and program B was framed in terms of risks wherein the probability and outcome of survival and death were known. Among those who received the version of the problem framed as gains or number of saved lives, most of the respondents chose the certain option. Conversely, more respondents preferred the risk-seeking option in the second version of the problem, which was framed as losses or number of casualties. From the perspective of a rational decision-making model, this can be considered irrational behavior, given that there is no substantial difference between the two versions [19,34]. In another example, McNeill et al. [35] showed that the probability of a group of fictitious patients choosing a survival frame on radiotherapy was substantially higher than a mortality frame. Specifically, their study participants were told that surgery could lead to immediate death in 10% of cases and short-term survival in 90% of cases.

Frames on topography and salience rest on the interaction between an organism’s learning history and the environment. Frames are not necessarily ways of acting upon the information itself, inasmuch as whether the salience of the information is perceived by the agent to whom it is directed. A stimulus is never intrinsically salient, and various factors can make stimuli or events “salient”. Generally, those factors fall into three categories: properties of the stimulus itself, how the stimulus fits with its context, and the internal cognitive state of the observer [36].

Throughout this work, we regard vaccine hesitancy as a product of different contingencies: (1) verbal contingencies (in particular, agents’ learning history) and (2) physical contingencies (e.g., salience, frames). We focus mainly on the second type of contingencies: specifically, we explore how highlighting different aspects of the data related to the effectiveness of COVID-19 can influence university students’ vaccine perceptions and their willingness to become vaccinated.

### 1.2. Experiment Rationale and Research Questions

The main problem in the spread of COVID-19 does not concern its contagion rate, per se, but, rather, the possible onset of severe forms of infection that require hospitalization. In fact, severe infections not only produce a high number of deaths, but they also significantly impact the resilience of national healthcare systems. This may be due to the fact that many infected users access these facilities concurrently, which may exceed the capacity of intensive care units.

Providing adequate information that produces greater adherence to vaccination campaigns is extremely important: specifically, this relates to how information is framed. The communication of potential protective effects as different types of COVID-19 vaccines started being mass-produced was framed in different ways, depending on communication channels and media. In Italy, some of them emphasized (i.e., increased salience on) the probability of reducing being infected by COVID-19; others emphasized the probability of avoiding hospitalization after being infected and experiencing severe symptoms.

In the present study, we aim to explore which of these two types of information framing positively affect perceptions towards vaccination and willingness to become vaccinated. The most common vaccines administered in Italy during the pandemic (i.e., Moderna, Pfizer-BioNTech, Johnson & Johnson, and AstraZeneca) featured a reduction in infection rate that ranged between 66% and 94%, which was lower than hospitalization rate (almost 100%). Thus, we expected that emphasizing vaccines’ protective aspects towards reducing the hospitalization rate is more effective than the infection rate.

The research questions on which this study rests are twofold. First, how can changes in supposedly irrelevant aspects of a problem affect the perceptions of vaccination based on information communication? Next, how can the data be communicated for positively affecting university students’ and other users’ willingness to become vaccinated? We postulate that it is possible to nudge vaccination rates in a positive direction by addressing the framing effects of protection and risks of getting vaccinated (or failing to). Addressing these research questions may provide valuable insights to policymakers for shaping more effective public health campaigns, especially targeting youths.

### 1.3. Hypotheses Formulation

We hypothesized that highlighting different aspects of the effectiveness of COVID-19 vaccines (i.e., reducing hospitalization rate vs. reducing infection rate) can influence people’s willingness to become vaccinated (main dimension) and the other five sub-dimensions related to vaccine perceptions: namely, reliability, trust, protection, safety, and confidence. Based on these general assumptions, we defined the following hypotheses.

Since the reduction of the rate of hospitalization was higher than the rate of infection, framing vaccine salience in terms of preventing hospitalization should increase willingness to become vaccinated compared to framing vaccine salience in terms of preventing infection. Thus, we expected that subjects exposed to the hospitalization prevention frame would provide higher scores of their willingness to become vaccinated than those exposed to the infection prevention frame (Hypothesis 1a). 

Moreover, willingness to become vaccinated should be more similar between vaccines when agents are exposed to information concerning vaccines’ efficacy in preventing hospitalization than infection. Thus, we hypothesized that willingness to become vaccinated with unbranded Vaccine 1 and Vaccine 2 types should be more similar when agents were exposed to the hospitalization prevention frame than the infection prevention frame (Hypothesis 1b). This expectation is based on the precondition that the data are always 100% in the hospitalization reduction rate, while there are two different data (66% and 94%) in the infection reduction rate.

Similar to willingness, vaccines’ efficacy in terms of preventing hospitalization should imply that agents have an overall better perception than in terms of preventing infection (Hypothesis 2a). In this sense, perceptions encompass reliability, trust, protection, safety, and confidence.

Finally, vaccines are hypothesized to be more similar in terms of each of the five sub-dimensions mentioned above when the vaccines’ efficacy is presented in terms of preventing hospitalization than preventing infection (Hypothesis 2b).

## 2. Methods

### 2.1. Participants and Setting

Data were collected in Italy in May 2021 among the students of three universities: two of these are located in Milan, and the third is in Enna. The sample consisted of 172 respondents who completed an online questionnaire developed on Survey Monkey. All participants who did not complete the questionnaire and those who failed the quality check were excluded from the statistical analysis. The first exclusion criterion was included to reduce the chance of participants selectively answering the questions in the questionnaire, undermining the reliability of the results. The latter was adopted to reduce data contaminations due to participants’ lack of understanding of the data in the experimental phase, rather than from their perceptions of the vaccines.

This left us with a final sample of N = 109 (M_age_ = 22.44, range 20–41 years old, 92.59% female). Within this sample, 15.6% of participants were vaccinated for COVID-19 with at least one dose, and 19.27% had recovered from COVID-19 at the time of data collection. Almost half of the participants (47.7%) reported being enrolled in an undergraduate degree, and about one-fifth (19.3%) earned a bachelor’s degree. For a detailed breakdown of participants’ demographics, please see the Supplementary File S1.

### 2.2. Experimental Design

We implemented a between-groups design that consisted of one independent variable and two independent groups. Participants were randomly assigned through the specific function embedded in Survey Monkey to one of two conditions: hospitalization prevention frame or infection prevention frame, which represented the independent variable. The dependent variable was the score provided by the participants on the dimensions included in the questionnaire.

### 2.3. Materials and Methods 

#### 2.3.1. Structure of the Questionnaire 

**Figure 1:**
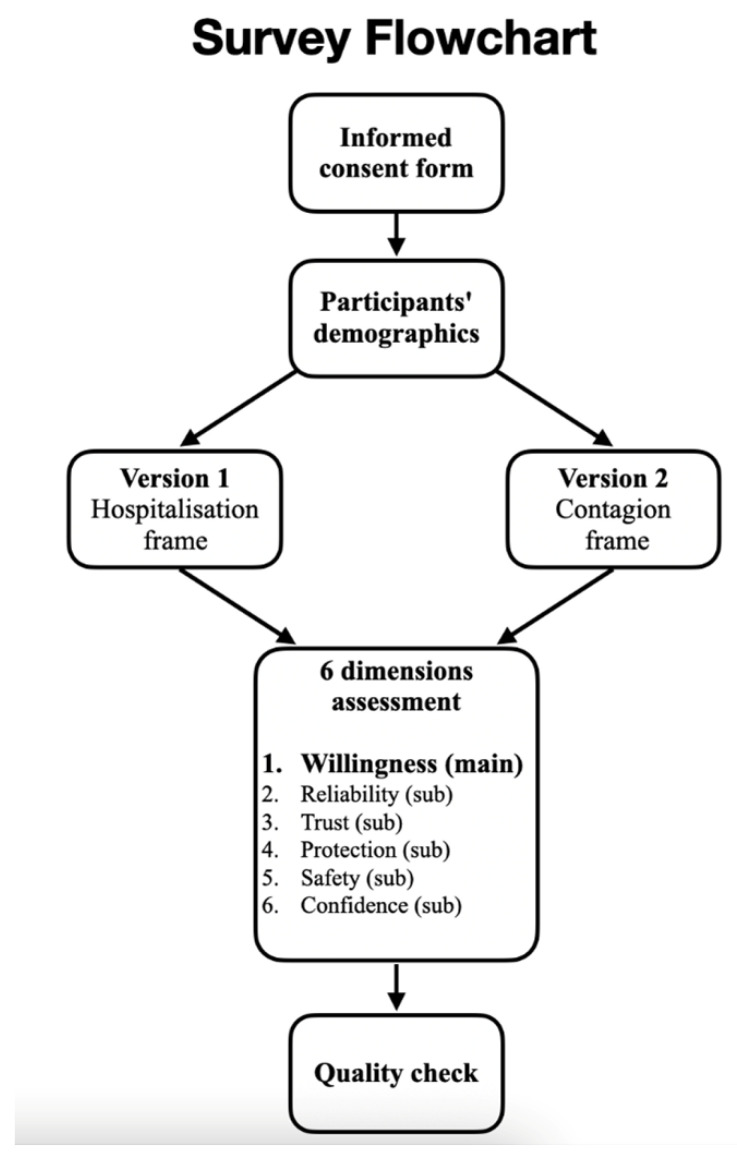
Overview of the six parts of the questionnaire. Note. Participants could withdraw at any time and were informed about this in the informed consent form. Please see the Section 2 for further detail.

The questionnaire unfolded in 6 main parts, whose structure is depicted in a flow-chart depicted in Figure 1. The structure of the questionnaire was defined through an ongoing process of revision that lasted about a month. During this phase, we focused on how to present the data about vaccine efficacy and the subsequent questions that would be useful for assessing the dimensions worth investigating. During that period, all the researchers involved in its development and other researchers and trainees from the entire IESCUM group completed pilot versions of the questionnaire several times. They provided feedback to improve its graphics and text in order to make it as comprehensible as possible for the participants.

Part 1 (informed consent). This part consisted of the informed consent form and a statement presented to all potential participants. Herein, all the information about the survey and the type of data that would be collected were presented. At the end of the statement, each participant was required to explicitly provide or deny their consent to using their data gathered through the questionnaire. If they denied their consent, they were sent to a landing page where they were praised for their time, and the survey was interrupted.

Part 2 (participants’ demographics). This part consisted of eleven items aimed at collecting participants’ demographics.

**Figure 2:**
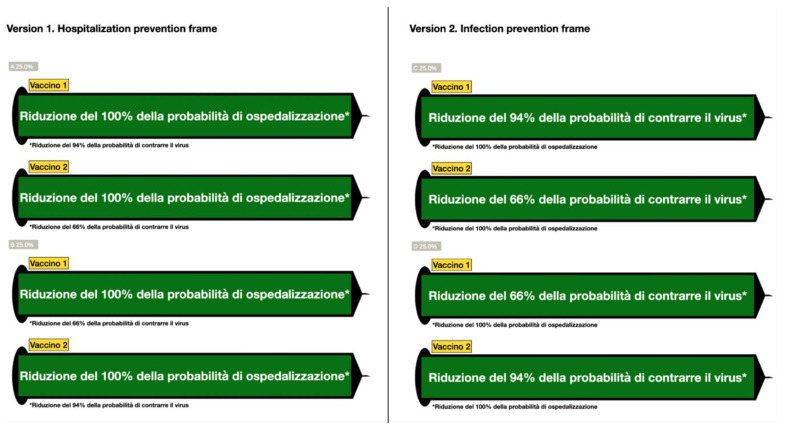
All four possible images displayed on the participants’ screen while completing the questionnaire. Note. English translation from Italian of the syringe texts on the left hand-side of the figure: “100% reduction in the probability of hospitalization; on the right hand-side: “94%/66% reduction in the probability of being infected with the virus”.

Part 3 (independent variable). This part included data on the vaccines and consisted of an image displaying information about the effectiveness of two vaccines to reduce hospitalization and infection in each condition. The information about the two vaccines was as follows: “Vaccine 1: 100% reduction of the probability of hospitalization; 94% reduction of the probability of being infected with the virus”. “Vaccine 2: 100% reduction in the probability of hospitalization; 66% reduction of the probability of being infected with the virus”. However, this information was arranged in different ways to make the data related to reducing the risks of hospitalization or infection more salient. Two versions of the data were provided, and each one unfolded into two subversions to balance possible order effects: these are illustrated in Figure 2 (The original is in Italian; a translation into English is provided in the note). Hence, participants were randomly assigned to one of four possible subversions.

In version 1 (subversions A and B), the data on reducing the risk of hospitalization were made more salient than the risk of infection. Conversely, in version 2 (subversions C and D), the data on the reduction in the risk of infection were made more salient than the risk of hospitalization.

Salience was manipulated in two main ways: (1) setting different font sizes so that the text displayed inside each syringe was twice as big as the text below the syringe, as well as (2) using a green background, filling each syringe to make the data contained in it more immediate and attractive to read. When answering the six assessment questions of participants’ learning history (i.e., the chance that they were answering based on previously learned information related to the vaccines used during the COVID-19 pandemic), we eliminated any reference to existing vaccine names, simply labeling the two vaccines as “Vaccine 1” and “Vaccine 2”.

Part 4 (dependent variable). This part consisted of six questions aimed at assessing one main dimension and five sub-dimensions related to participants’ attitudes and perceptions towards each of the two vaccines. The process that led to the definition of the main dimension and the five sub-dimensions consisted of several brainstorming meetings and focus groups, involving the project leader, IESCUM researchers and trainees, and all other researchers involved in the design of the experiment. This process allowed for convergence on several dimensions what were deemed to be of particular relevance for adhering to national vaccination campaigns. The order of questions for assessing the six dimensions was randomized among the participants. The dimensions and their related questions translated from Italian into English were as follows.

Willingness (main dimension). This dimension aimed to investigate how willing study participants were to become vaccinated based on the following question: “Based on the observed data, how willing are you to get each of the following vaccines?” The question was followed by a rating scale ranging from 1 to 10, where 1 meant “not willing at all” and 10 meant “completely willing”.

Reliability (sub-dimension). This dimension aimed to investigate how reliable study participants considered each vaccine based on the following question: “Based on the observed data, how reliable do you think each of the following vaccines is?”. The question was followed by a rating scale ranging from 1 to 10, where 1 meant “not reliable at all”, and 10 meant “completely reliable”.

Trust: (sub-dimension). This dimension aimed to investigate how much study participants trusted each vaccine in terms of their capacity to reduce possible health issues and based on the following question: “Based on the observed data, how much do you trust each of the following vaccines?”. The question was followed by a rating scale, ranging from 1 to 10, where 1 meant “no trust at all”, and 10 meant “trust completely”.

Protection (sub-dimension). This dimension aimed to investigate how protected participants felt by each vaccine based on the following question: “Based on the observed data, how protected would you feel from COVID-19 if you were given each of the following vaccines?”. The question was followed by a rating scale, ranging from 1 to 10, where 1 meant “not protected at all”, and 10 meant “completely protected”.

Safety (sub-dimension). This dimension aimed to investigate how safe participants believed each vaccine was based on the following question: “Based on the observed data, how safe would you feel to get each of the following vaccines?”. The question was followed by a rating scale ranging from 1 to 10, where 1 meant “not safe at all”, and 10 meant “completely safe”.

Confidence (sub-dimension). This dimension aimed to investigate how confident participants felt about getting each vaccine based on the following question: “Based on the observed data, how confident would you feel about getting vaccinated with each of the following vaccines?”. The question was followed by a rating scale ranging from 1 to 10, where 1 meant “not confident at all”, and 10 meant “completely confident”.

Part 5 (control item). This part consisted of one question for checking whether participants understood what the data referred to before completing the questions on the six dimensions. The item included the following statement: “To ensure that you read carefully the data that we showed you, please indicate below what the percentages regarding vaccines effectiveness referred to”. Next, participants were asked to select one of four possible response options (among which only answer number 2 was correct), namely: (1) reduction in the risk of hospitalization and thrombosis; (2) reduction in the risk of hospitalization and infection with the virus; (3) reduction in the risk of infection with the virus and thrombosis; and (4) none of the above. 

Part 6 (debriefing item). This part consisted of an open-ended textbox provided to the participants at the end of the survey to allow them to write comments or provide feedback regarding the completion of the questionnaire. The text included in this item read as follows: “The questionnaire is now complete; thank you for participating! Before closing the form, you can leave a comment, note, or feedback in the box below”.

#### 2.3.2. Procedure

The questionnaire was shared with the students by email or intranet, introducing it with the following text: “Dear Student, some fellow researchers of IULM University are conducting a short survey on issues related to the current health emergency. We would like to ask for your availability to spend 5 min of your time to fill it out. To do so, you will have to connect to this link. The data collected in the questionnaire will be completely anonymous and used in aggregate form. You will be able to stop filling out the questionnaire at any time. Thanks in advance for your collaboration”.

Once the participants clicked on the link, they were readdressed to the questionnaire home page, which contained the consent form. After they expressed their informed consent (part 1) and provided the required demographic information (part 2), the experimental part of the questionnaire started.

At first, each participant was presented with the following text: “The image below presents data on currently available vaccines for COVID-19 prevention. Please look at the data carefully; later, you will be asked to give your opinion on some dimensions related to the presented vaccines. Take as much time as you feel necessary to explore the data, as you cannot go back to view the image. Once you have looked at the data, click on the green “next” text box at the bottom to continue filling out this questionnaire”. Below the text, we showed one of the four possible images, reproduced in Figure 2.

Next, participants were required to answer six questions for assessing the dimensions described above (part 4). Once they filled out the central part of the questionnaire, they were asked to answer a control item (part 5). Finally, they were presented with the debriefing item at the end of the questionnaire (part 6). There were no time constraints for completing the questionnaire.

#### 2.3.3. Ethical Considerations

In addition to the explicit collection of participants’ informed consent before starting the data collection, all data were collected anonymously. This entailed that we did not record sensitive data that could directly or indirectly (e.g., tracking IP addresses) be linked to a single respondent’s answers. Regarding informed consent, participants were instructed that they could stop filling out the questionnaire at any time.

In part 2 of the questionnaire (i.e., participants’ demographics), the option “I prefer not to answer” was included for items that investigated more personal aspects of the participants (e.g., their political orientation, whether COVID-19 has infected them or their relatives, or whether they have either booked their vaccination appointment or gotten their vaccine dose).

#### 2.3.4. Analysis

Using a multilevel linear regression model, we tested the effect of the interaction between the variables vaccine type (Vaccine 1 vs. Vaccine 2) and condition (hospitalization frame vs. infection prevention frame) on each of the six dimensions measured in the questionnaire. The model specified the variables vaccine type, condition, and the two-way interaction between vaccine type and condition as predictors, together with gender and age as covariates. In order to take into account the repeated measure design, we allowed random intercepts for the variable subject. We performed the analysis using the lmerTest R package (version 3.1–3) [37]. All the multilevel models were estimated using Restricted Maximum Likelihood (REML), *p*-values were calculated based on Satterthwaite’s approximations, and confidence intervals were calculated using the profile likelihood method. All the analyses that were performed are available in the supplementary material.

## 3. Results

For all the assessed dimensions, the covariate age did not correlate significantly with the dependent variable. Furthermore, there was no statistically significant difference in ratings between male and female participants. Overall, participants reported that they were more willing to becom vaccinated with Vaccine 1 compared to Vaccine 2.

### 3.1. Main Dimension

**Figure 3:**
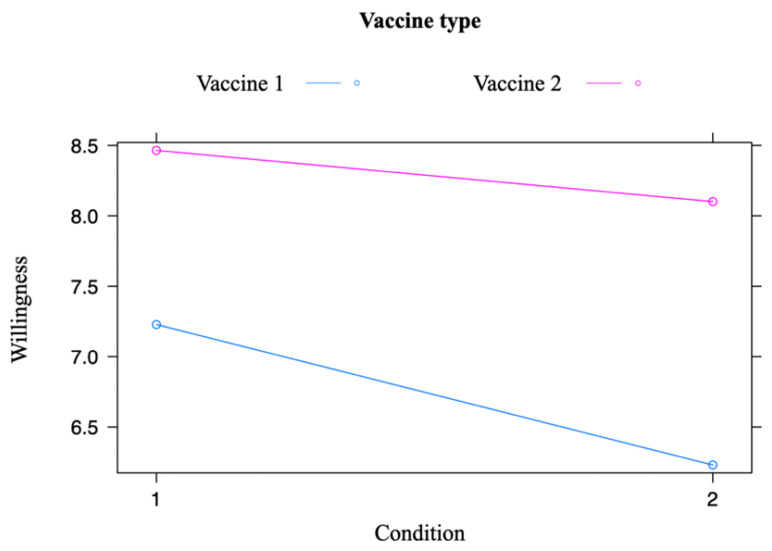
Willingness to become vaccinated, based on framing condition. Note. abscissa: condition 1 refers to the hospitalization frame, and condition 2 to the infection prevention frame.

By taking into account the main dimension of the study (i.e., willingness to get vaccinated), the predictor condition had a main effect on the dependent variable. In general, participants reported being more willing to become vaccinated when exposed to the hospitalization frame than the infection prevention frame (B = −0.91, 95% CI [−1.83, 0.02], t(140.48) = −1.91, *p* = 0.037). The effect of interaction between the variables vaccine type and condition was not statistically significant, although the difference in the evaluation of willingness to vaccinate with either Vaccine 1 or Vaccine 2 tended towards significance when participants were exposed to the infection frame compared to the hospitalization frame (B = 0.63, 95% CI [−0.10, 1.36], t(107) = 1.70, *p* = 0.091) (see also Figure 3).

### 3.2. Sub-Dimensions

By taking into account the five sub-dimensions concerning the predictor condition, we found a statistically significative effect only of the sub-dimension confidence (B = −0.88, 95% CI [−1.76, 0.01], t(132.43) = −1.92, *p* = 0.044). In contrast, we found an interaction effect between vaccine type and condition of the sub-dimensions reliability (B = 0.63, 95% CI [0.04, 1.22], t(107) = 2.11, *p* = 0.037), safety (B = 0.72, 95% CI [0.11, 1.33], t(107) = 2.30, *p* = 0.024), and confidence (B = 0.87, 95% CI [0.24, 1.49], t(107) = 2.71, *p* = 0.008). 

## 4. Discussion

This study aimed to evaluate the effects of stimulus salience manipulation by framing different forms of the same information regarding the efficacy of COVID-19 vaccines on willingness and perceptions towards vaccination. Willingness to become vaccinated was chosen as the main dimension, as it was thought to be the most closely related to public health policymakers’ target behavior during the pandemic (i.e., getting as many as possible vaccinated). In addition, the effect of different frames was explored on five additional sub-dimensions related to vaccine perception: reliability, trust, protection, safety, and confidence.

Based on an overall review of research hypotheses and results, we found that it is possible to influence, to some extent, university students’ attitudes and perceptions by altering how the information on each vaccine was framed. In line with the expressed stated intentions of this work, our findings translated into promoting better practices for individual and public health, such as vaccinating against COVID-19.

Considering the willingness to become vaccinated (main dimension), respondents exposed to the hospitalization frame condition provided higher scores than those exposed to the infection frame condition. Hence, Hypothesis 1a was confirmed. Regarding Hypothesis 1b, the rate of being willing to become vaccinated with Vaccine 1 and Vaccine 2 tended to be more similar when respondents were exposed to the hospitalization frame condition than when they were exposed to the infection frame condition.

Hypotheses 2a and 2b considered the effect of frames on the remaining five sub-dimensions of this study (i.e., reliability, trust, protection, safety, and confidence). While we expected to find similar effects to those reported for the main dimension, we found mixed results.

Confidence was a particularly informative sub-dimension, as there was both a main effect and an interaction effect of category. Previous studies highlighted the importance of confidence on determining vaccine hesitancy. For example, in the “3 Cs” model proposed by the WHO EURO Working Group on vaccine communications in 2011, “confidence is defined as trust in (i) the effectiveness and safety of vaccines; (ii) the system that delivers them, including the reliability and competence of the health services and health professionals and (iii) the motivations of policy-makers who decide on the needed vaccines” [3]. It is not possible to identify, from our data, which of these sources of trust or mistrust exerted the highest control on this sample of young university students in Italy. Our interpretation is that confidence is a multifaceted term that prompts different reactions, based on the respondents’ learning history (i.e., whatever event that affected the behavior throughout the ontogenesis of an agent) and environment. Moreover, these results are consistent with previous research that associated COVID-19 vaccine hesitancy and “lack of trust/confidence in scientists, healthcare personnel, health institutions and/or the government” [38].

Interestingly, no main nor interaction effect was observed on the sub-dimension trust, although it has been previously pointed out as the single key dimension for overcoming vaccine hesitancy (along with fighting misinformation) [39]. In a comprehensive systematic review, trust was strongly correlated with vaccine acceptance (R = 0.78, *p* < 0.01) [40]. Because “trust is built with consistency” (L. Chafee), policymakers should shape build communication campaigns on transparency of intentions, evidence-based interventions, and two-way feedback with end-users and other stakeholders. Although this may differ from the Italian context, trust in government among youths was found to be generally low in a sample of secondary students in Hong Kong; thus, this affects their willingness to become vaccinated and our society’s preparedness against future pandemics, staring from its younger members [41].

The present study fits in with and continues the long tradition of research that emerged from the stream of studies focused on the so-called framing effects, pioneered in the 1980s by Tversky and Kahneman, e.g., [19,32]. From a traditional economic view, framing represents an irrelevant aspect of a problem. The invariance principle states that agents’ choices should not be influenced by how the same information is presented. However, several findings within the research domain of behavioral economics point out how differences in formal aspects of the same information affect a decision response, see also [32]. We embraced the latter perspective and showed the potential of frame manipulation for increasing willingness to become vaccinated. Previous studies have explored the effects of other types of frames on intentions to vaccinate. For example, Hong and Hashimoto [42] tested gain/loss and self/others’ reference points, depending on high/low infection risk, and they found that possible adverse effects among significant others had exerted the most robust effect among young adults (i.e., self-perceived low risk).

The findings of the study should be considered in light of its limitations. First, our results warrant being interpreted with caution because of the small sample size. Moreover, 25 respondents (about 15% of the sample size) failed to correctly answer the control item, which may call for several explanations. Beside possible distraction effects, misunderstanding of the data, and constrains due to time or effort, respondents may have been primed into answering thrombosis based on communication campaigns and news, highlighting that risky consequence while the experiment was running. This effect may be described in terms of heuristics and biases: for example, this may be communicated in terms of availability and primacy of information on side effects. On the other hand, we believe that the inclusion of a control item represents a strength of our data collection procedure, insofar as it filtered out a relatively high share of possible data contamination.

Secondly, the dimension chosen as the main target of the study (i.e., willingness to become vaccinated) does not allow direct inferences to be made about the actual choice to become vaccinated. Therefore, it seems essential for future research to demonstrate to what extent willingness translates into policymakers’ target behavior: supposedly, increasing vaccination rates and adhesion to prophylaxis for containing the virus spread and mortality. For example, possible concerns with communication campaigns included legitimizing public discussions of vaccine hesitancy (i.e., a self-fulfilling prophecy), which did not empirically supported [43]. Another important barrier to agents’ willingness to become vaccinated is presented by conspiracy beliefs, which were significantly associated with lower vaccination intentions [44].

Third, the dimensions examined in the study were not subject to a formal process of validation. While developing the survey, the researchers and trainees involved in its design repeatedly discussed and revised the questions to minimize the chance of possible divergent interpretations of the dimensions. However, we cannot exclude the possibility that participants still perceived them differently from what the researchers hypothesized.

Finally, we resorted to a convenience sampling method of a young segment of the population drawn from university students with a higher education level compared to other segments. For example, according to a recent report published by Italy’s National Institute of Statistics (ISTAT), only 62.7% (cf. all the participants in our study) of the Italian population aged between 25 and 64 possesses at least a high school diploma [45]. It is possible that the participants in our study had different attitudes and perceptions than older segments of the population or segments that possess a lower (or higher) level of education. Although our sample was not representative of any of these segments, nor does it allow us to generalize our results, we may advance some considerations, based on previous research, that shares some characteristics (i.e., aim and sample) with the present work. For example, the study of Hong and Hashimoto [42] supports the idea of framing vaccination as prosocial behavior, rather than individual health behavior, to increase the willingness to vaccinate among young university students, who rate themselves and, in fact, are less likely to suffer from the same severe symptoms that characterize other age groups.

Despite these limitations, our results have several conceptual and practical implications. First, our findings provide valuable insights for improving the communication strategies of any agency responsible for disseminating information or news to the public. Contingently, differential communication strategies can influence the choices of news recipients based on marketers’ aims and how they convey information to the specific groups or the broader public. Considering the potential effect of framing information on agents’ decisions can help designing communication campaigns that are more effective at promoting recipients’ compliance with health guidelines, this can be performed while significantly impacting social and economic costs. Current knowledge about individual decision-making processes makes it possible to design communication strategies and behavior modification interventions informed by behavioral science, e.g., see [46].

In several cases, the result was chosen to disseminate information on reducing the infection rate if the goal was to reduce the hospitalization rate. This was especially true, considering the reduction in the hospitalization rate was substantial and characterized by low variability between vaccines. For example, while the communication of risks of COVID-19 on adults has tended to focus on infecting others, the intention to vaccinate children among a sample of UK respondents doubled when the focus was on the risks of vaccination for the children themselves compared to the local community [47,48]. 

## 5. Conclusions

Our work consists of an exploratory study with a limited sample size of university students in Italy. Nevertheless, it has enhanced our understanding of the relationship between information framing, as well as willingness and perceptions towards vaccination in this segment of the population and provided some indications for stimulating further research efforts on vaccine hesitancy, which is key to policymaking and public health.

Our findings add to the body of evidence that can inform policymakers regarding the communication of medical risks and influence the vaccination choice in the context of the COVID-19 pandemic and beyond. Based on their questionnaire results on COVID-19 vaccine hesitancy, Ebrahimi et al. [49] concluded that responding to affective reactions and involving community leaders are the most promising remedies to hesitancy. Hesitancy also decreased in terms of risk perception among potential flu vaccine recipients following the COVID pandemic’s reopening phase [50]. Interventions should not be limited to summing up the behavior of the individuals of which our communities and society are composed; they should also include a level of selection represented by the culture, which addresses cultural practices that may be transmitted more efficiently and with enduring results see [51].

We maintain that our approach above seems particularly valuable for policymaking and public health. Specifically, this study has the potential and ambition of informing behaviorally informed interventions for improving current choice repertoires. Potentially, it increases the likelihood that these repertoires are transmitted further in time and across agents and agencies through their selection at the cultural and community level.

## Data Availability

The data supporting this study’s findings are available from the corresponding author upon reasonable request.

## Supplementary Materials

The following supporting information can be downloaded at: https://www.mdpi.com/article/10.3390/vaccines11061079/s1, Supplementary File S1: statistical analyses.
