# Supplementary material for: Framing Effects on Willingness and Perceptions towards COVID-19 Vaccination among University Students in Italy: An Exploratory Study
Source: Vaccines (Basel). 2023 Jun 9;11(6):1079. doi: 10.3390/vaccines11061079 (PMC10301223; doi:10.3390/vaccines11061079)
Supplement: Supplementary file 1 [file vaccines-11-01079-s001.zip › vaccines-2410379-supplementary.pdf]

Analisi dei dati studio Covid-19 e vaccinazioni

6/30/2021

N= 109

Analisi descrittive delle variabili demografiche

Media e deviazione standard dell'età sul campione aggregato e sui tre campioni separatamente

1= IULM, 2= Sigmund Freud University, 3= Kore

```
pander(summary(data$eta)); pander(sd(data$eta))
```

Min.	1st Qu.	Median	Mean	3rd Qu.	Max.
20	21	22	22.44	23	41

2.891

```
eta.desc<- summarySE(data, measurevar="eta", groupvars=c("raccolta_dati"), na.rm=TRUE)
pander(eta.desc)
```

raccolta_dati	N	eta	sd	se	ci
1	52	21.44	1.018	0.1411	0.2833
2	43	23.4	3.36	0.5125	1.034
3	14	23.21	4.611	1.232	2.662

Frequenze per la variabile genere sul campione aggregato e sui tre campioni separatamente

```
data$genere<- as.factor(data$genere)
pander(summary(data$genere))
```

Campione aggregato

Altro	F	M
1	100	8

```
iulm$genere<- as.factor(iulm$genere)
pander(summary(iulm$genere))
```

IULM

F	M
50	2

```r
sfu$genere<- as.factor(sfu$genere)
pander(summary(sfu$genere))
```

Sigmund Freud University

Altro	F	M
1	37	5

```r
kore$genere<- as.factor(kore$genere)
pander(summary(kore$genere))
```

Kore

F	M
13	1

Media e SD dell'orientamento politico nel campione aggregato e nei tre campioni separatamente

1= IULM, 2= Sigmund Freud University, 3= Kore

-4 = estrema sinistra; +4= estrema destra

```r
pander(summary(data$orientamento_politico)); pander(sd(data$orientamento_politico))
```

Min.	1st Qu.	Median	Mean	3rd Qu.	Max.	NA's
-3	-3	-2	-1.355	-1	4	47

NA

```r
orientamento.desc<- summarySE(data, measurevar="orientamento_politico", groupvars=c("raccolta_dati"), na
pander(orientamento.desc)
```

raccolta_dati	N	orientamento_politico	sd	se	ci
1	32	-1.406	1.434	0.2534	0.5169
2	24	-1.292	1.732	0.3534	0.7312
3	6	-1.333	2.251	0.9189	2.362

Frequenze per la variabile educazione sul campione aggregato e sui tre campioni separatamente

1= medo di un diploma di scuola superiore; 2= diploma di scuola superiore o equivalente; 3= Università, senza conseguire la laurea; 4= Laurea triennale; 5= Laurea magistrale; 6= diploma di specializzazione

```r
data$educazione<- as.factor(data$educazione)
pander(summary(data$educazione))
```

Campione aggregato

2	3	4	5
34	52	21	2

```r
iulm$educazione<- as.factor(iulm$educazione)
pander(summary(iulm$educazione))
```

IULM

2	3	4
22	27	3

```r
sfu$educazione<- as.factor(sfu$educazione)
pander(summary(sfu$educazione))
```

Sigmund Freud University

2	3	4	5
9	14	18	2

```r
kore$educazione<- as.factor(kore$educazione)
pander(summary(kore$educazione))
```

Kore

2	3
3	11

Frequenze per la variabile occupazione sul campione aggregato e sui tre campioni separatamente

1= full time; 2= part time; 3= studente; 4= pensionato; 5= disoccupato; 6= altro

```r
data$occupazione<- as.factor(data$occupazione)
pander(summary(data$occupazione))
```

Campione aggregato

1	2	3	5	6
2	5	99	2	1

```r
iulm$occupazione<- as.factor(iulm$occupazione)
pander(summary(iulm$occupazione))
```

IULM

1	2	3
1	3	48

```r
sfu$occupazione<- as.factor(sfu$occupazione)
pander(summary(sfu$occupazione))
```

Sigmund Freud University

1	2	3	5	6
1	1	39	1	1

```r
kore$occupazione<- as.factor(kore$occupazione)
pander(summary(kore$occupazione))
```

Kore

2	3	5
1	12	1

Frequenze per la variabile residenza sul campione aggregato e sui tre campioni separatamente

1= Italia; 2= Estero

```r
data$residenza<- as.factor(data$residenza)
pander(summary(data$residenza))
```

Campione aggregato

1	2
105	4

```r
iulm$residenza<- as.factor(iulm$residenza)
pander(summary(iulm$residenza))
```

IULM

1	2
50	2

```
sfu$residenza<- as.factor(sfu$residenza)
pander(summary(sfu$residenza))
```

Sigmund Freud University

1	2
41	2

```
kore$residenza<- as.factor(kore$residenza)
pander(summary(kore$residenza))
```

Kore

1
14

Frequenze per la variabile fonte di informazione

1= si, ho usato questa fonte di informazione; 2= no, non ho usato questa fonte di informazione

Campione aggregato

```
data$info_social<- as.factor(data$info_social)
pander(summary(data$info_social))
```

Social

0	1
47	62

```
data$info_TV<- as.factor(data$info_TV)
pander(summary(data$info_TV))
```

TV

0	1
38	71

```
data$info_giornali<- as.factor(data$info_giornali)
pander(summary(data$info_giornali))
```

Giornali

0	1
96	13

```
data$info_internet<- as.factor(data$info_internet)
pander(summary(data$info_internet))
```

Internet

0	1
25	84

```
data$info_medico<- as.factor(data$info_medico)
pander(summary(data$info_medico))
```

Medico curante

0	1
102	7

```
data$info_persone<- as.factor(data$info_persone)
pander(summary(data$info_persone))
```

Persone vicine

0	1
72	37

Frequenze per la variabile ho fatto il faccino

1 = sì, appartengo a una delle categorie professionali per cui è stata prevista la vaccinazione; 2 = sì, appartengo a una delle categorie di popolazione fragile/vulnerabile; 3 = no, non ho intenzione di farlo; 4 = no, non ho ancora avuto la possibilità di farlo; 5 = preferisco non rispondere

```
data$vaccino<- as.factor(data$vaccino)
pander(summary(data$vaccino))
```

Campione aggregato

1	2	3	4	5
4	13	8	82	2

```
iulm$vaccino<- as.factor(iulm$vaccino)
pander(summary(iulm$vaccino))
```

IULM

2	3	4	5
2	4	45	1

```r
sfu$vaccino<- as.factor(sfu$vaccino)
pander(summary(sfu$vaccino))
```

Sigmund Freud University

1	2	3	4	5
4	7	3	28	1

```r
kore$vaccino<- as.factor(kore$vaccino)
pander(summary(kore$vaccino))
```

Kore

2	3	4
4	1	9

Frequenze per la variabile ho contratto il covid

1 = no; 2 = si, con sintomatologia lieve; 3 = si, con sintomatologia severa (es. necessità di ossigeno a domicilio, necessità di ricovero); 4 = preferisco non rispondere

```r
data$covid<- as.factor(data$covid)
pander(summary(data$covid))
```

Campione aggregato

1	2	3
88	20	1

```r
iulm$covid<- as.factor(iulm$covid)
pander(summary(iulm$covid))
```

IULM

1	2
44	8

```r
sfu$covid<- as.factor(sfu$covid)
pander(summary(sfu$covid))
```

Sigmund Freud University

1	2	3
32	10	1

```r
kore$covid<- as.factor(kore$covid)
pander(summary(kore$covid))
```

Kore

1	2
12	2

Frequenze per la variabile le persone vicine a me hanno contratto il covid

1 = no; 2 = si, con sintomatologia lieve; 3 = si, con sintomatologia severa (es. necessità di ossigeno a domicilio, necessità di ricovero); 4 = preferisco non rispondere

```r
data$covid_contatti<- as.factor(data$covid_contatti)
pander(summary(data$covid_contatti))
```

Campione aggregato

1	2	3
18	55	36

```r
iulm$covid_contatti<- as.factor(iulm$covid_contatti)
pander(summary(iulm$covid_contatti))
```

IULM

1	2	3
7	30	15

```r
sfu$covid_contatti<- as.factor(sfu$covid_contatti)
pander(summary(sfu$covid_contatti))
```

Sigmund Freud University

1	2	3
9	16	18

```r
kore$covid_contatti<- as.factor(kore$covid_contatti)
pander(summary(kore$covid_contatti))
```

Kore

1	2	3
2	9	3

Frequenze per la variabile condizione

1 = salienza ospedalizzazioni; 2 = salienza contagi

```r
data$condizione<- as.factor(data$condizione)
pander(summary(data$condizione))
```

Campione aggregato

1	2
55	54

```r
iulm$condizione<- as.factor(iulm$condizione)
pander(summary(iulm$condizione))
```

IULM

1	2
28	24

```r
sfu$condizione<- as.factor(sfu$condizione)
pander(summary(sfu$condizione))
```

Sigmund Freud University

1	2
22	21

```r
kore$condizione<- as.factor(kore$condizione)
pander(summary(kore$condizione))
```

Kore

1	2
5	9

Test delle ipotesi

Campione aggregato

Modello:

DV ~ vaccino * condizione + genere + + (1|id)

Ipotesi:

Dovrebbe esserci un effetto significativo (p< .05) dell'interazione vaccino * condizione, cioè la variabile dipendente dovrebbe variare a seconda del vaccino valutato (66% cotro 94%) e della condizione di salienza (ospedalizzazione contro contagi) a cui il partecipante è stato sottoposto

```
protetto.mod<-lmer(valutazione_protetto ~ vaccino * condizione + genere + (1|id), data=data.protetto)
pander(anova(protetto.mod))
```

VD: quanto ti sentiresti protetto

Table 43: Type III Analysis of Variance Table with Satterthwaite's method

	Sum Sq	Mean Sq	NumDF	DenDF	F value	Pr(>F)
**vaccino**	216.6	216.6	1	107	147.5	7.401e-22
**condizione**	1.387	1.387	1	105	0.9446	0.3333
**genere**	3.098	1.549	2	105	1.055	0.3519
**vaccino:condizione**	4.406	4.406	1	107	3.001	0.08609

```
summary(protetto.mod)
```

```
## Linear mixed model fit by REML. t-tests use Satterthwaite's method [
## lmerModLmerTest]
## Formula: valutazione_protetto ~ vaccino * condizione + genere + (1 | id)
##    Data: data.protetto
##
## REML criterion at convergence: 845.6
##
## Scaled residuals:
##      Min       1Q   Median       3Q      Max
## -2.79458 -0.50541  0.01277  0.53095  1.92804
##
## Random effects:
##  Groups   Name        Variance Std.Dev.
##  id       (Intercept) 2.137    1.462
##  Residual             1.468    1.212
## Number of obs: 218, groups:  id, 109
##
## Fixed effects:
##                              Estimate Std. Error       df t value Pr(>|t|)
## (Intercept)                    8.1455     1.6982 105.9764   4.796 5.31e-06 ***
## vaccinovaccino_94              1.7091     0.2311 107.0000   7.397 3.30e-11 ***
## condizione2                   -0.6020     0.3658 154.9546  -1.646   0.1018
## genereF                       -1.6293     1.7103 105.0000  -0.953   0.3429
## genereM                       -2.3014     1.8086 105.0000  -1.272   0.2060
## vaccinovaccino_94:condizione2  0.5687     0.3283 107.0000   1.732   0.0861 .
```

```
## ---
## Signif. codes:  0 '***' 0.001 '**' 0.01 '*' 0.05 '.' 0.1 ' ' 1
##
## Correlation of Fixed Effects:
##             (Intr) vcc_94 cndzn2 generF generM
## vccnvccn_94 -0.068
## condizione2 -0.021  0.316
## genereF     -0.988  0.000 -0.084
## genereM     -0.935  0.000 -0.101  0.939
## vccnvc_94:2  0.048 -0.704 -0.449  0.000  0.000
```

- Le persone si sentono generalmente più protette all'idea di essere vaccinati con il vaccino 94% (p< .001).
- L'effetto dell'interazione vaccino * condizione tende alla significatività (p= .086), mostrando che, mentre le valutazioni del vaccino 94% restano invariate a seconda della condizione sperimentale, le valutazioni del vaccino 66% sono più basse nella condizione di salienza contagi rispetto alla condizione di salienza ospedalizzazione (vedere figura sotto)

```
plot(Effect(c("condizione", "vaccino"), protetto.mod), "condizione", "vaccino", multiline = T)
```

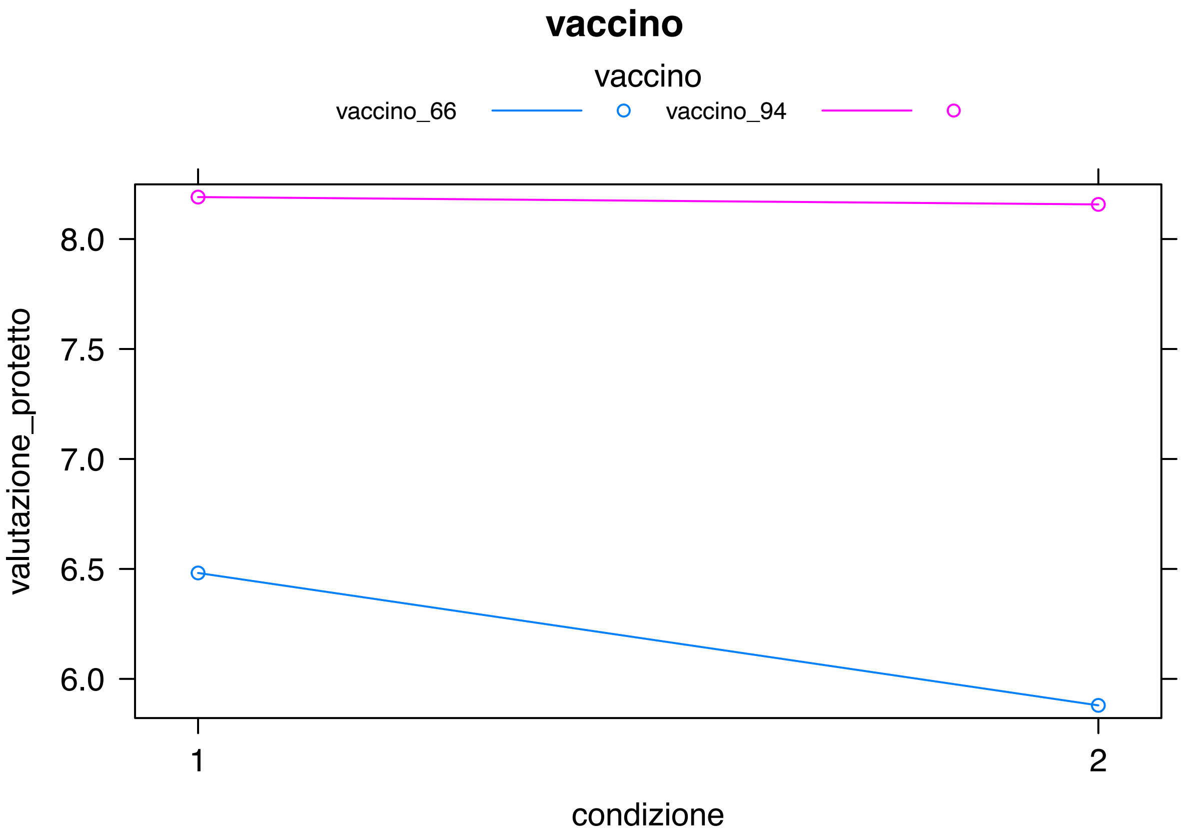

VD: quanto ritieni affidabile

```
affidabile.mod<-lmer(valutazione_affidabile ~ vaccino * condizione + genere + (1|id), data=data.affidabi
pander(anova(affidabile.mod))
```

Table 44: Type III Analysis of Variance Table with Satterthwaite's method

	Sum Sq	Mean Sq	NumDF	DenDF	F value	Pr(>F)
**vaccino**	110.6	110.6	1	107	90.47	6.508e-16
**condizione**	0.09999	0.09999	1	105	0.08176	0.7755

	Sum Sq	Mean Sq	NumDF	DenDF	F value	Pr(>F)
**genere**	2.096	1.048	2	105	0.857	0.4274
**vaccino:condizione**	5.436	5.436	1	107	4.445	0.03735

```
summary(affidabile.mod)
```

```
## Linear mixed model fit by REML. t-tests use Satterthwaite's method [
## lmerModLmerTest]
## Formula: valutazione_affidabile ~ vaccino * condizione + genere + (1 |      id)
##    Data: data.affidabile
##
## REML criterion at convergence: 838.1
##
## Scaled residuals:
##      Min       1Q   Median       3Q      Max
## -2.29727 -0.49547  0.02619  0.45599  2.16609
##
## Random effects:
##  Groups   Name        Variance Std.Dev.
##  id       (Intercept) 2.610    1.615
##  Residual             1.223    1.106
## Number of obs: 218, groups:  id, 109
##
## Fixed effects:
##                              Estimate Std. Error       df t value Pr(>|t|)
## (Intercept)                    8.4455     1.7979 105.7248   4.697 7.96e-06 ***
## vaccinovaccino_94              1.1091     0.2109 107.0000   5.259 7.47e-07 ***
## condizione2                   -0.4148     0.3773 143.0636  -1.100   0.2734
## genereF                       -1.7365     1.8117 105.0000  -0.958   0.3400
## genereM                       -2.3131     1.9159 105.0000  -1.207   0.2300
## vaccinovaccino_94:condizione2   0.6316     0.2996 107.0000   2.108   0.0373 *
## ---
## Signif. codes:  0 '***' 0.001 '**' 0.01 '*' 0.05 '.' 0.1 ' ' 1
##
## Correlation of Fixed Effects:
##             (Intr) vcc_94 cndzn2 generF generM
## vccnvccn_94 -0.059
## condizione2 -0.016  0.279
## genereF     -0.989  0.000 -0.086
## genereM     -0.935  0.000 -0.104  0.939
## vccnvc_94:2  0.041 -0.704 -0.397  0.000  0.000
```

- Le persono valutano come più affidabile il vaccino al 94% (p< .001).
- L'interazione vaccino * condizione è significativa (p = .037), mostrando che, mentre le valutazioni del vaccino 94% sono più alte nella condizione di salienza contagi rispetto alla condizione di salienza ospedalizzazione, le valutazioni del vaccino 66% sono più basse nella condizione di salienza contagi rispetto alla condizione di salienza ospedalizzazione (vedere figura sotto). Da notare che i coefficienti delle due curve non sono significativi (i beta non sono diversi da zero), ma la significatività dell'interazione è dovuta al fatto che i coefficienti hanno segno diverso (i beta sono diversi tra loro) (vedere figura sotto).

```r
plot(Effect(c("condizione", "vaccino"), affidabile.mod), "condizione", "vaccino", multiline = T)
```

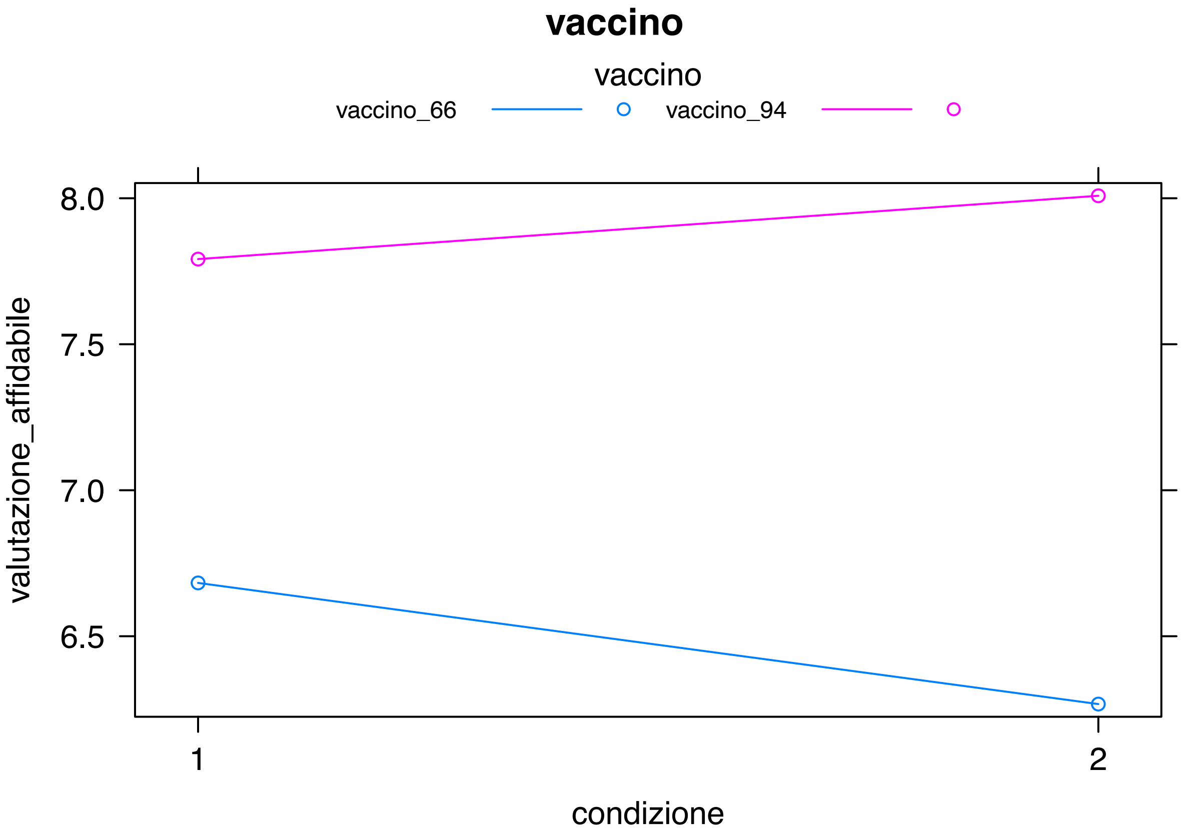


```r
disponibilita.mod<-lmer(valutazione_disponibilita ~ vaccino * condizione + genere + (1|id), data=data.di
pander(anova(disponibilita.mod))
```

VD: quanto saresti disponibile a farti vaccinare

Table 45: Type III Analysis of Variance Table with Satterthwaite's method

	Sum Sq	Mean Sq	NumDF	DenDF	F value	Pr(>F)
**vaccino**	131.5	131.5	1	107	69.65	2.716e-13
**condizione**	4.589	4.589	1	105	2.431	0.122
**genere**	6.836	3.418	2	105	1.811	0.1686
**vaccino:condizione**	5.476	5.476	1	107	2.901	0.09145

```r
summary(disponibilita.mod)
```

```
## Linear mixed model fit by REML. t-tests use Satterthwaite's method [
## lmerModLmerTest]
## Formula: valutazione_disponibilita ~ vaccino * condizione + genere + (1 |
##     id)
##    Data: data.disponibilita
##
## REML criterion at convergence: 933.2
```

```
##
## Scaled residuals:
##      Min       1Q   Median       3Q      Max
## -2.39809 -0.37017 -0.01948  0.57916  2.06298
##
## Random effects:
##  Groups   Name        Variance Std.Dev.
##  id       (Intercept) 4.173    2.043
##  Residual             1.888    1.374
## Number of obs: 218, groups:  id, 109
##
## Fixed effects:
##                              Estimate Std. Error       df t value Pr(>|t|)
## (Intercept)                    9.3818     2.2659 105.7044   4.140 6.99e-05 ***
## vaccinovaccino_94              1.2364     0.2620 107.0000   4.719 7.22e-06 ***
## condizione2                   -0.9974     0.4745 142.0581  -2.102   0.0373 *
## genereF                       -2.0716     2.2834 105.0000  -0.907   0.3664
## genereM                       -3.4497     2.4148 105.0000  -1.429   0.1561
## vaccinovaccino_94:condizione2   0.6340     0.3723 107.0000   1.703   0.0914 .
## ---
## Signif. codes:  0 '***' 0.001 '**' 0.01 '*' 0.05 '.' 0.1 ' ' 1
##
## Correlation of Fixed Effects:
##             (Intr) vcc_94 cndzn2 generF generM
## vccnvccn_94 -0.058
## condizione2 -0.016  0.276
## genereF     -0.989  0.000 -0.086
## genereM     -0.935  0.000 -0.104  0.939
## vccnvc_94:2  0.041 -0.704 -0.392  0.000  0.000
```

- Le persone sarebbero più disponibili a farsi vaccinare con il vaccino al 94% ($p< .001$), e sarebbero generalmente più disponibili a farsi vaccinare le persone esposte alla condizione disalienza delle ospedalizzazioni ($p= .037$).
- L'interazione vaccino * condizione tende alla significatività ($p= .091$), mostrando che l'effetto della condizione sperimentale è maggiore per il vaccino 66% rispetto al vaccino 94%. In altre parole, mentre le valutazioni del vaccino 94% differivano poco tra condizioni sperimentali, le valutazioni del vaccino 66% variavano maggiormente tra condizioni sperimentali di salienza contagi versus ospedalizzazioni, con disponibilità più bassa a farsi vaccinare nella condizione di salienza dei contagi (vedere figura sotto).

```r
plot(Effect(c("condizione", "vaccino"), disponibilita.mod), "condizione", "vaccino", multiline = T)
```

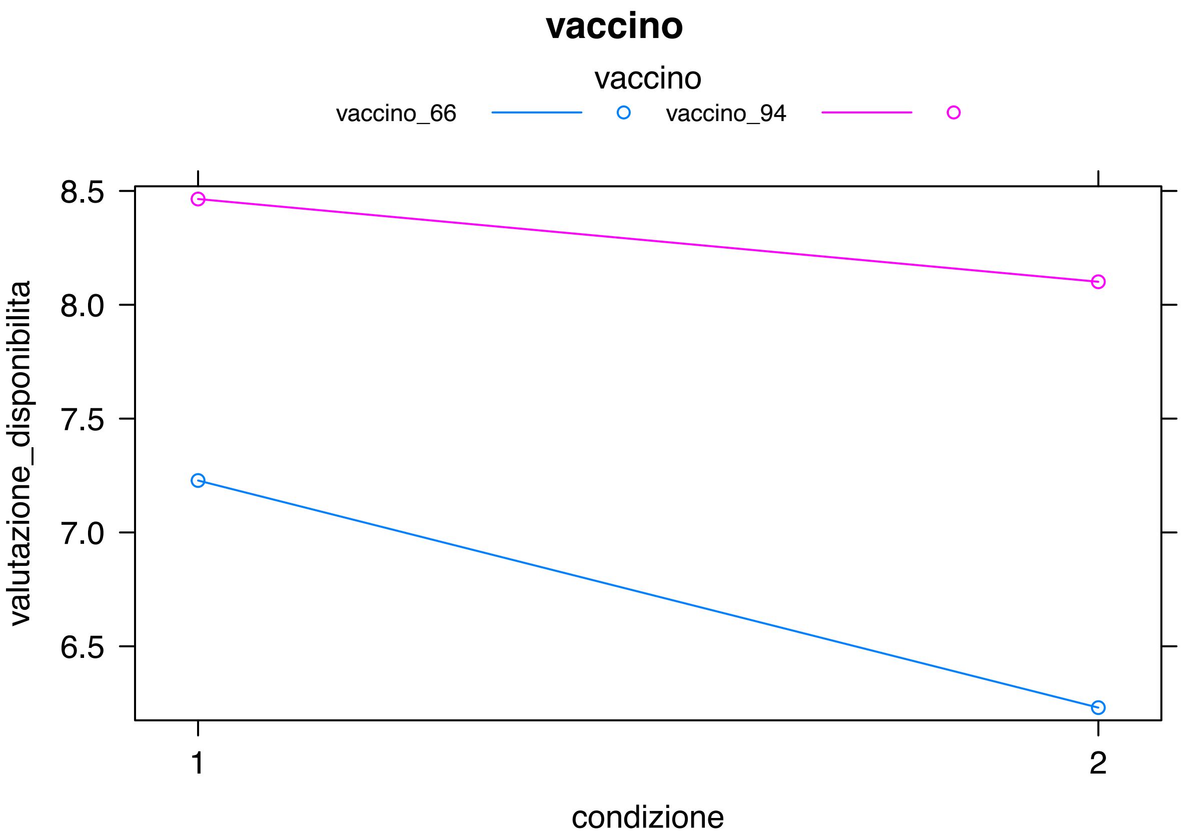


```
fiducia.mod<-lmer(valutazione_fiducia ~ vaccino * condizione + genere + (1|id), data=data.fiducia)
pander(anova(fiducia.mod))
```

VD: quanto ti fidi

Table 46: Type III Analysis of Variance Table with Satterthwaite's method

	Sum Sq	Mean Sq	NumDF	DenDF	F value	Pr(>F)
**vaccino**	88.93	88.93	1	107	61.11	3.997e-12
**condizione**	1.893	1.893	1	105	1.301	0.2567
**genere**	2.909	1.454	2	105	0.9994	0.3716
**vaccino:condizione**	3.167	3.167	1	107	2.176	0.1431

```
summary(fiducia.mod)
```

```
## Linear mixed model fit by REML. t-tests use Satterthwaite's method [
## lmerModLmerTest]
## Formula: valutazione_fiducia ~ vaccino * condizione + genere + (1 | id)
##    Data: data.fiducia
##
## REML criterion at convergence: 879.9
##
## Scaled residuals:
##      Min       1Q   Median       3Q      Max
```

```
## -2.82621 -0.38333  0.01259  0.42049  2.54678
##
## Random effects:
##  Groups   Name        Variance Std.Dev.
##  id       (Intercept) 3.288    1.813
##  Residual             1.455    1.206
## Number of obs: 218, groups:  id, 109
##
## Fixed effects:
##                                Estimate Std. Error       df t value Pr(>|t|)
## (Intercept)                      8.4818     2.0072 105.6919   4.226 5.07e-05 ***
## vaccinovaccino_94                1.0364     0.2300 107.0000   4.505 1.70e-05 ***
## condizione2                     -0.6820     0.4197 141.4382  -1.625    0.106
## genereF                         -1.6290     2.0228 105.0000  -0.805    0.422
## genereM                         -2.4744     2.1391 105.0000  -1.157    0.250
## vaccinovaccino_94:condizione2   0.4822     0.3268 107.0000   1.475    0.143
## ---
## Signif. codes:  0 '***' 0.001 '**' 0.01 '*' 0.05 '.' 0.1 ' ' 1
##
## Correlation of Fixed Effects:
##             (Intr) vcc_94 cndzn2 generF generM
## vccnvccn_94 -0.057
## condizione2 -0.016  0.274
## genereF     -0.989  0.000 -0.086
## genereM     -0.935  0.000 -0.104  0.939
## vccnvc_94:2  0.040 -0.704 -0.389  0.000  0.000
```

- Le persone si fidano di più del vaccino al 94%, e questo è indipendente dall'effetto della condizione di salienza a cui sono state sottoposte.
- Nessun effetto dell'interazione vaccino * condizione.

```
ritieni.sicuro.mod<-lmer(valutazione_ritieni_sicuro ~ vaccino * condizione + genere + (1|id), data=data
pander(anova(ritieni.sicuro.mod))
```

VD: quanto ritieni sicuro

Table 47: Type III Analysis of Variance Table with Satterthwaite's method

	Sum Sq	Mean Sq	NumDF	DenDF	F value	Pr(>F)
**vaccino**	75.57	75.57	1	107	56.62	1.74e-11
**condizione**	0.2143	0.2143	1	105	0.1606	0.6894
**genere**	3.292	1.646	2	105	1.233	0.2956
**vaccino:condizione**	7.04	7.04	1	107	5.275	0.02358

```
summary(ritieni.sicuro.mod)
```

```
## Linear mixed model fit by REML. t-tests use Satterthwaite's method [
## lmerModLmerTest]
## Formula: valutazione_ritieni_sicuro ~ vaccino * condizione + genere +
##     (1 | id)
##    Data: data.ritieni.sicuro
##
```

```
## REML criterion at convergence: 875.8
##
## Scaled residuals:
##      Min       1Q   Median       3Q      Max
## -2.97242 -0.42948 -0.00311  0.56812  2.62198
##
## Random effects:
##  Groups   Name        Variance Std.Dev.
##  id       (Intercept) 3.549    1.884
##  Residual             1.335    1.155
## Number of obs: 218, groups:  id, 109
##
## Fixed effects:
##                               Estimate Std. Error       df t value Pr(>|t|)
## (Intercept)                     6.3151     0.7749 109.3294   8.149 6.58e-13 ***
## vaccinovaccino_94               0.8182     0.2203 107.0000   3.714 0.000326 ***
## condizione2                    -0.5182     0.4260 137.0497  -1.217 0.225884
## genereAltro                     3.2758     2.1920 105.0000   1.494 0.138067
## genereF                         0.7136     0.7564 105.0000   0.943 0.347636
## vaccinovaccino_94:condizione2   0.7189     0.3130 107.0000   2.297 0.023580 *
## ---
## Signif. codes:  0 '***' 0.001 '**' 0.01 '*' 0.05 '.' 0.1 ' ' 1
##
## Correlation of Fixed Effects:
##             (Intr) vcc_94 cndzn2 gnrAlt generF
## vccnvccn_94 -0.142
## condizione2 -0.334  0.259
## genereAltro -0.346  0.000  0.105
## genereF     -0.922  0.000  0.066  0.326
## vccnvc_94:2  0.100 -0.704 -0.367  0.000  0.000
```

- Le persone tendono valutano il vaccino 94% come più sicuro (p< .001).
- L'interazione vaccino * condizione è significativa (p= .023), mostrando che, le persone esposte alla condizione di salienza dei contagi ritengono più sicuro il vaccino a 94% e meno sicuro il vaccino al 66% rispetto alle persone nella condizione di salienza delle ospedalizzazioni. In altre parole, nella condizione di salienza contagi la differenza tra le valutazioni dei vaccini e maggione rispetto alla differenza tra le valutazioni dei vaccini nella condizione di salienza delle ospedalizzazioni (vedere figura sotto).

```r
plot(Effect(c("condizione", "vaccino"), ritieni.sicuro.mod), "condizione", "vaccino", multiline = T)
```

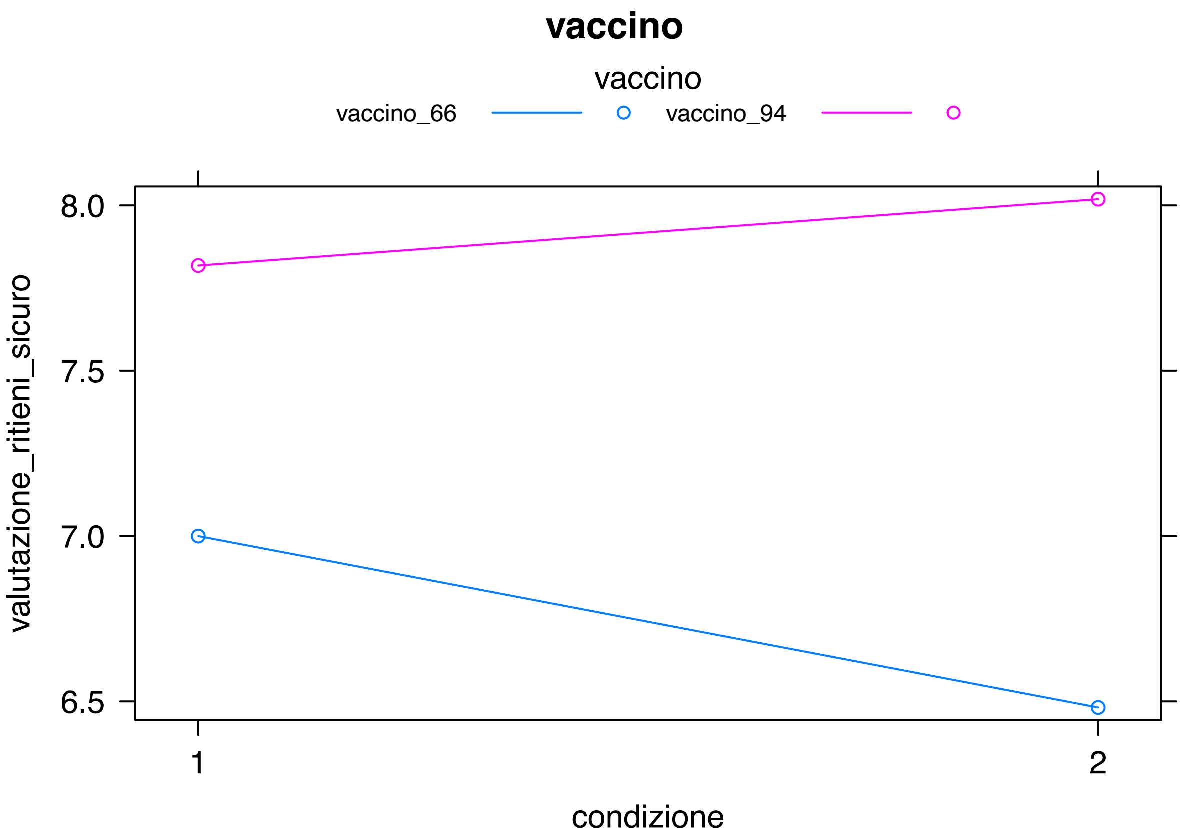


```
ritieni.sicuro.mod<-lmer(valutazione_sentiresti_sicuro ~ vaccino * condizione + genere + (1|id), data=da
pander(anova(ritieni.sicuro.mod))
```

VD: quanto ti sentiresti sicuro

Table 48: Type III Analysis of Variance Table with Satterthwaite's method

	Sum Sq	Mean Sq	NumDF	DenDF	F value	Pr(>F)
**vaccino**	82.89	82.89	1	107	59.37	7.044e-12
**condizione**	1.863	1.863	1	105	1.334	0.2507
**genere**	5.043	2.522	2	105	1.806	0.1694
**vaccino:condizione**	10.23	10.23	1	107	7.329	0.0079

```
summary(ritieni.sicuro.mod)
```

```
## Linear mixed model fit by REML. t-tests use Satterthwaite's method [
## lmerModLmerTest]
## Formula: valutazione_sentiresti_sicuro ~ vaccino * condizione + genere +
##     (1 | id)
##    Data: data.sentiresti.sicuro
##
## REML criterion at convergence: 895.2
##
## Scaled residuals:
```

```
##      Min       1Q   Median       3Q      Max 
## -2.81830 -0.46763 -0.04892  0.50616  2.54150 
## 
## Random effects:
##  Groups   Name        Variance Std.Dev.
##  id       (Intercept) 4.146    2.036   
##  Residual             1.396    1.182   
## Number of obs: 218, groups:  id, 109
## 
## Fixed effects:
##                              Estimate Std. Error       df t value Pr(>|t|)    
## (Intercept)                    7.2253     0.3232 133.5560  22.358  < 2e-16 ***
## vaccinovaccino_94              0.8000     0.2253 107.0000   3.550 0.000573 ***
## condizione2                   -0.9238     0.4538 134.2963  -2.036 0.043747 *  
## genereAltro                    2.3747     2.2218 105.0000   1.069 0.287610    
## genereM                       -1.2563     0.8107 105.0000  -1.550 0.124261    
## vaccinovaccino_94:condizione2   0.8667     0.3201 107.0000   2.707 0.007900 ** 
## ---
## Signif. codes:  0 '***' 0.001 '**' 0.01 '*' 0.05 '.' 0.1 ' ' 1
## 
## Correlation of Fixed Effects:
##             (Intr) vcc_94 cndzn2 gnrAlt generM
## vccnvccn_94 -0.349                            
## condizione2 -0.689  0.248                     
## genereAltro -0.128  0.000  0.088              
## genereM     -0.139  0.000 -0.066  0.020       
## vccnvc_94:2  0.245 -0.704 -0.353  0.000  0.000
```

- Le persone si sentirebbero più sicure a farsi vaccinare con il vaccino al 94% (p< .001).
- Le persone nella condizione di salienza ospedalizzazioni si sentirebbero più sicure a farsi vaccinare (p= .044).
- L'interazione vaccino * condizione è significative (p= .008), mostrando che, mentre le valutazioni del vaccino 94% non cambiano a seconda della condizione sperimentale di salienza a cui le persone sono state sottoposte, le valutazioni del vaccino 66% sono più basse nella condizione di salienza dei contagi rispetto alla condizione di salienza ospedalizzazioni (vedere figura sotto)

```r
plot(Effect(c("condizione", "vaccino"), ritieni.sicuro.mod), "condizione", "vaccino", multiline = T)
```

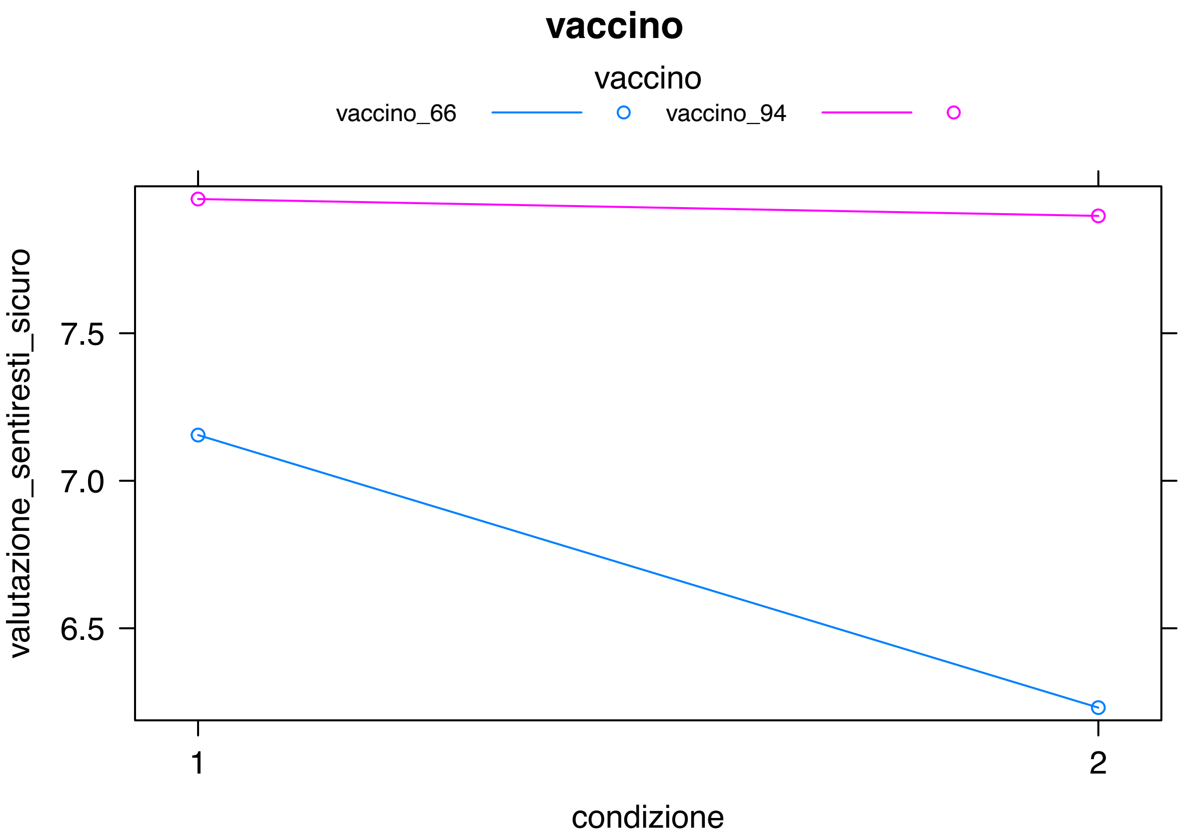
vaccino
vaccino
vaccino_66
vaccino_94
valutazione_sentiresti_sicuro
7.5
7.0
6.5
1
2
condizione
